# Serial sarcomere adaptation to eccentric overload training is blunted in ovariectomized rats

**DOI:** 10.14814/phy2.71070

**Published:** 2026-08-24

**Authors:** Amelia Rilling, Alexandra Q. Kirkup, Avery Hinks, Martino V. Franchi, Geoffrey A. Power

**Affiliations:** ^1^ Department of Human Health and Nutritional Sciences College of Biological Sciences, University of Guelph Guelph Ontario Canada; ^2^ Department of Biomedical Sciences University of Padova Padova Italy

**Keywords:** eccentric overload, estradiol, fascicle, longitudinal remodeling, muscle architecture, sarcomerogenesis

## Abstract

Eccentric contractions performed to long muscle lengths impose high mechanical stress and strain on the muscle fiber, providing a stimulus for longitudinal growth (i.e., increases in serial sarcomeres number (SSN)). Conventional (CONV) resistance training incorporates eccentric and concentric phases; however, mechanical loading is constrained by the concentric phase. Eccentric overload (ECC_OVERLOAD_) training has a maximal load throughout the eccentric phase increasing the potential for greater sarcomerogenesis. The purpose was to investigate ECC_OVERLOAD_ and CONV training on mechanical and morphological adaptation in ovary‐intact and OVX rats. Intact and OVX Sprague–Dawley rats performed 4 weeks (×3/week) of ECC_OVERLOAD_ or CONV resistance training. Mechanical measures were tested pre‐ and post‐training. Fascicle length and sarcomere length were measured to obtain SSN. Torque increased (*p* < 0.001) similarly across groups at the shorter muscle (*p* = 0.075) and greater at the longer muscle lengths (*p* = 0.002) in OVX rats. In the soleus, there was a training× hormone status× training group (*p* = 0.028) interaction, such that intact rats showed the greatest improvement in SSN with ECC_OVERLOAD_ (~13%), which was higher than both CONV intact (~3%) and OVX rats in either training condition (ECC_OVERLOAD_ ~1%, CONV ~5%). Therefore, OVX had a blunted adaptation in longitudinal muscle remodeling in response to ECC_OVERLOAD_ training.

## 1 INTRODUCTION

The arrangement of sarcomeres in a muscle influences its contractile performance (Hinks et al., 2023; Lieber & Ward, 2011), such that when sarcomeres are added in parallel (i.e., radial growth), maximal force production is increased (Goldspink, 1985; Jorgenson et al., 2020). Whereas, an increase in serially aligned sarcomeres (i.e., longitudinal growth) can result in faster shortening velocity and a broadening of the force‐length relationship's plateau region, increasing the muscle's force production potential throughout a wider range of motion and improving power output (Hinks et al., 2023; Lieber & Ward, 2011).

Eccentric contractions performed to long muscle lengths impose high mechanical stress and strain on the muscle, providing a potent stimulus for longitudinal muscle growth (i.e., increases in serial sarcomere number (SSN)) (Chen et al., 2020; Hinks et al., 2022; Hinks et al., 2023; Hinks, Patterson, et al., 2024; Rilling et al., 2026). However, eccentric contractions are rarely performed in isolation during conventional resistance training, where an eccentric contraction occurs following a concentric contraction. The muscle's force‐producing capacity during eccentric contractions (i.e., lowering phase) far exceeds that of concentric contractions (i.e., lifting phase), resulting in a submaximal eccentric phase (Kowalchuk & Butcher, 2019), which may not provide a strong enough mechanical loading environment for longitudinal muscle adaptations to occur (Butterfield et al., 2005a; Hinks et al., 2023; Rilling et al., 2026). Comparatively, eccentric overload training, where the stimulus is maximized throughout the eccentric phase, increases stress and strain (Tøien et al., 2018) on the muscle promoting greater longitudinal remodeling (Rilling et al., 2026). Eccentric training has been shown to increase serial sarcomere number (SSN) in young (Butterfield et al., 2005a; Chen et al., 2020; Hinks et al., 2022; Lynn et al., 1998) and old healthy male rats (Hinks, Patterson, et al., 2024), as well as humans (Andrews et al., 2025). Recently, we showed that eccentric overload training resulted in greater sarcomerogenesis than conventional resistance training in adult male and female rats with a 17% increase in SSN of the soleus (Rilling et al., 2026). Interestingly, the increase in SSN with eccentric overload training was primarily driven by female rats (Rilling et al., 2026), leading us to suspect there may be sex‐specific differences in training induced sarcomerogenesis driven by sex‐hormones.

Estradiol is a primary female sex‐hormone which augments muscle recovery and lessens initial eccentric exercise‐induced damage through inflammation attenuation (Tiidus, 2003) and initiation of satellite cell repair processes (Collins, Laakkonen, & Lowe, 2019; Enns & Tiidus, 2008; Moran et al., 2006; Seko et al., 2020; Tiidus, 2003). In estradiol deficient females there are decreased satellite cell density, blunted repair and greater muscle damage following acute injury (Collins, Laakkonen, & Lowe, 2019; Enns & Tiidus, 2008; Seko et al., 2020). Additionally, estradiol may upregulate signaling pathways which are involved in sarcomerogenesis and pertinent to cell growth and survival. As serial sarcomerogenesis is likely driven by mechanical stress/strain of the muscle at longer lengths, estradiol may alleviate the initial detrimental effects of eccentric exercise and help facilitate recovery and ultimately enhance the longitudinal remodeling response (Rilling et al., 2026). Conversely, estradiol deficiency may increase cytotoxic neutrophil accumulation (You et al., 2022) and blunt repair processes potentially hindering functional remodeling and ultimately reducing the muscle's ability to add sarcomeres in series.

Currently, there has been no investigation into the influence of estradiol deficiency on eccentric training‐induced sarcomerogenesis. However, serial sarcomerogenesis induced through repetitive stretching (10 × 1‐min static stretches with 45‐s rest intervals for 3 weeks) in OVX and ovary‐intact rats demonstrates an increase (~15%–20%; SSN) in intact rats and a negligible increase in OVX rats (Vidal et al., 2020). As well, the same stretching protocol for 6 weeks demonstrated 8% greater increases in soleus SSN in ovary‐intact compared to OVX rats (Ywazaki et al., 2016). Additionally, it has been shown that the pelvic floor muscles of rats undergo sarcomerogenesis before parturition (Rieger et al., 2022). The pre‐parturition sarcomerogenesis was caused by a combined effect of both increased loading of the pelvic floor and the shifting hormonal milieu of pregnancy, highlighting the potential modulatory role of sex‐hormones on sarcomerogenesis (Rieger et al., 2022). That said, the effect of estradiol deficiency on SSN and mechanical adaptations to conventional and eccentric overload resistance training remains unknown.

The purpose of this study was twofold: (1) To investigate the effects of eccentric overload training compared to conventional resistance training on SSN in female rats, (2) to determine whether ovariectomy and its associated changes in the hormonal milieu influence the magnitude of training‐induced serial sarcomerogenesis. We hypothesized that: (1) eccentric overload training will yield a greater magnitude of SSN increase than conventional resistance training; and (2) OVX rats will have a blunted response to eccentric overload training compared to the ovary‐intact rats.

## 2 MATERIALS AND METHODS

### 2.1 Animals

Forty (*N* = 40; *N* = 20 female controls [age = 13–17 weeks; mass = 259.25 ± 22.54], *N* = 20 OVX [age = 14–17 weeks; mass = 290.85 ± 29.24]) female Sprague–Dawley rats were obtained (Charles River Laboratories, QC, Canada). Rats were housed in groups of two or three at 23°C and given a Teklad global 18% protein rodent diet (Envigo, Huntington, Cambs., UK) and water ad‐libitum. To adhere to the 3R's of animal research, a subset of ovary intact rats (*n* = 9; *n* = 5 ECC_OVERLOAD_; *n* = 4 CONV) were reused from a prior study (Rilling et al., 2026), however, entirely new fascicles were teased out and measured for both sarcomere length and fascicle length to derive new SSN. All protocols were approved by the University of Guelph's Animal Care Committee (AUP #4905). Baseline pre‐training mechanical testing was completed at ~12–14 weeks of age then training commenced ~3 days later, occurring 3 days/week (Monday, Wednesday, Friday) for 4 weeks and 48–72 h following the final training session, post‐training mechanical testing was conducted. All training and mechanical testing was conducted on the left leg with the right leg acting as an internal control for muscle wet mass, anatomical and physiological cross‐sectional area (ACSA and PCSA, respectively), and SSN in accordance with previous studies (de la Tour et al., 1979; Heslinga & Huijing, 1993; Hinks, Patterson, et al., 2024; Kinney et al., 2017; Williams & Goldspink, 1978). The soleus and medial gastrocnemius (MG) muscles were dissected for SSN determinations on both legs (Hinks, Patterson, et al., 2024; Williams & Goldspink, 1978).

### 2.2 Ovariectomy procedure

Ovariectomized female rats were obtained from Charles River Laboratories (Charles River Laboratories, QC, Canada), where all surgical procedures were performed by trained staff under the oversight of their Institutional Animal Care and Use Committee (IACUC) prior to arrival at our facility. Animals were ≥21 days old at the time of surgery and received standard pre‐ and postoperative care according to Charles River guidelines. For the surgical procedure, a small dorsal incision was made parallel to the midline, and the skin was retracted to expose the underlying abdominal wall. The abdominal cavity was entered via a blunt puncture, and the ovary and associated fat pad were exteriorized. The fallopian tube was cauterized, the ovary was removed, and the remaining fat pad and tissue were returned to the abdominal cavity. The procedure was then repeated on the contralateral side. The skin incision was closed using wound clips, which were removed 7–10 days following surgery. Approximately 2–4 weeks following ovariectomy, estradiol levels have significantly declined to minimally detectable levels (Rodríguez‐Landa, 2022). Postoperatively, animals were held for a minimum of 24 h, with most shipments occurring within 7 days of surgery. The ovariectomy model results in a decrease in estradiol, progesterone, and testosterone (Bonilla‐Becerra et al., 2017; Chang et al., 2019). An important consideration in this model is that the loss in estradiol causes a luteinizing hormone (LH) and follicle‐stimulating hormone rise (FSH); (Yen & Tsai, 1971) (see discussion). A methodological consideration of the present study is that there was no sham operated group to control for any stress or inflammatory response due to the surgery rather than ovariectomy alone. We mitigated the adverse surgery effects by waiting at least 2 weeks following ovariectomy to begin testing. Additionally, all morphological measures are within rat and therefore occurred almost 2 months following surgery. Furthermore, while no serum estradiol levels were taken, ovariectomy has been shown to drastically decrease estradiol levels (Chang et al., 2019).

### 2.3 Mechanical testing data acquisition

Transcutaneous muscle stimulation was evoked using a 701C High‐Powered, Bi‐Phase Stimulator (Aurora Scientific, ON, Canada). A custom‐made electrode holder with steel galvanized pins was placed transversely over the popliteal fossa and the calcaneal tendon. Torque, angle, and stimulus trigger data were sampled at 1000 Hz with a 605A Dynamic Muscle Data Acquisition and Analysis System (Aurora Scientific, ON, Canada). A live torque trace was created during all training and mechanical testing sessions.

### 2.4 Mechanical testing

**FIGURE 1:**
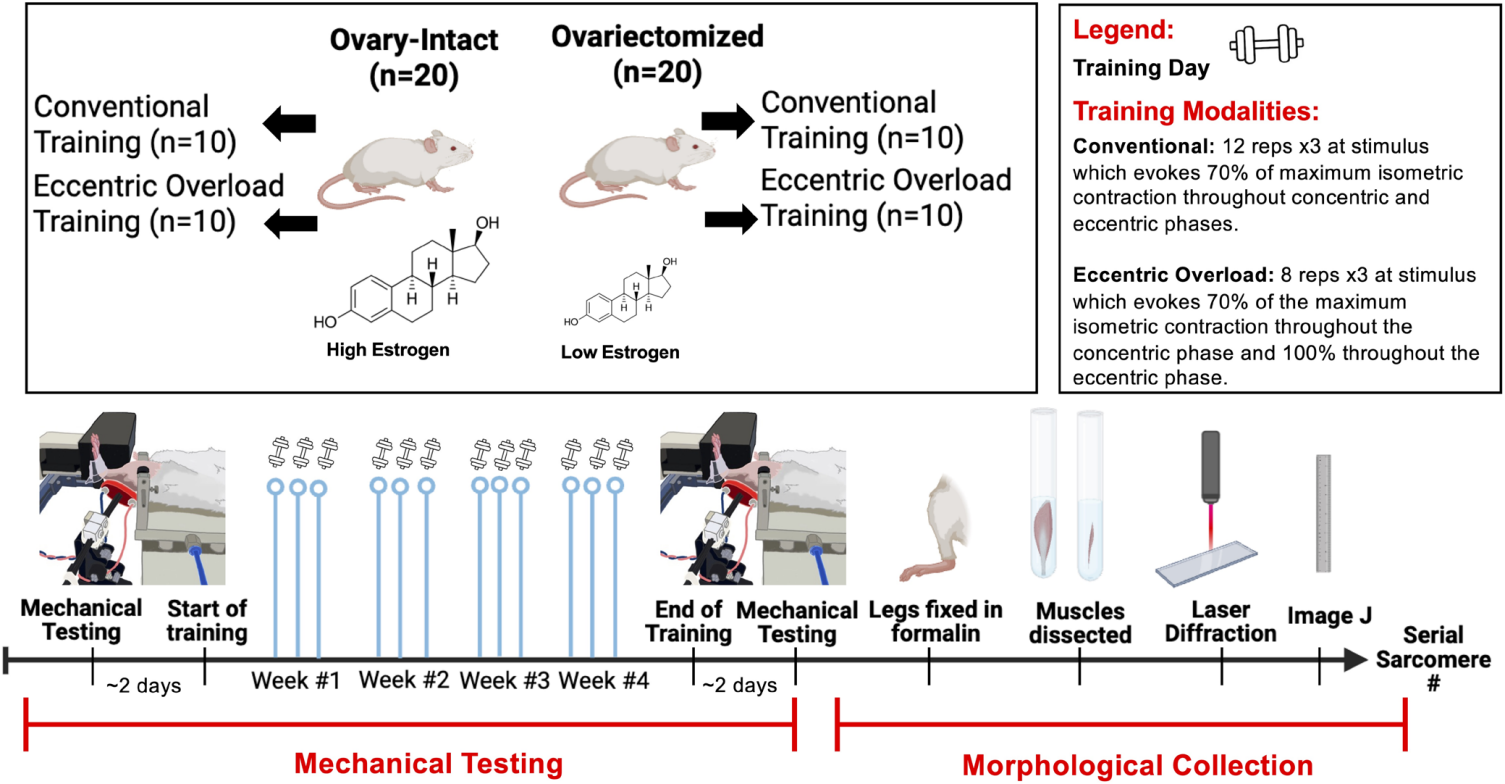
Experimental timeline demonstrating the mechanical testing, training and morphological data collection. Female rats (*n* = 40; 20 ovary intact, 20 OVX) underwent 4 weeks of eccentric overload (*n* = 20) or conventional resistance training (*n* = 20). The ovary‐intact group represented the high estrogen condition, and the OVX group represented the low estrogen condition. Mechanical testing was done pre and post 4 weeks of training. Rats were anesthetized and placed supine on a heated platform (37°C) with a nose cone providing isoflurane. The left leg was secured with a leg clamp around the knee (150°), with the left foot secured with tape to a foot plate attached to a torque motor/transducer. Percutaneous stimulation electrodes were positioned over the plantar flexors as well as the tibial nerve to evoke contractions. The ankle is pictured at 90° with a range of motion of 70°–110°.

Isoflurane was used to anesthetize all rats, and they were placed on a 37°C platform in a supine position. The rat's left leg was shaved, and the leg was placed and secured in a force transducer/length controller with a clamp near the knee and the foot was fixed with tape to a foot pedal (Figure 1). Mechanical testing sessions began with the determination of the stimulation current for maximal active plantar flexion torque (frequency = 100 Hz, pulse duration = 0.1 ms, train duration = 500 ms) at an ankle angle of 90° (full plantar flexion = 180°). The identified current was transmitted through the custom‐made electrode and used throughout the remainder of the testing session. A 100 Hz stimulation was then completed at an ankle angle of 70°. To determine the active torque, the baseline (passive torque) was subtracted from the maximum total torque value during stimulation (Wisdish et al., 2025). Passive torque at an ankle angle of 90° and 70° was recorded to gain insight on adaptations in muscle passive properties. A torque‐angular velocity‐power relationship was established from isotonic contractions. Each isotonic contraction began at 70° and rotated to a joint angle of 110° against a fixed load clamp. The isotonic load clamps equated to 10%, 20%, 30%, 40%, 50%, 60%, 70%, and 80% of the maximum torque produced at a 70‐degree ankle angle. The order of the isotonic contractions was randomized using an automated randomizer application. Angular velocity was recorded as the average slope of the position‐time trace during shortening. Two minutes of rest separated each stimulation to minimize the development of muscle fatigue.

The estimated maximum shortening velocity at zero load (V_max_), maximum power (torque multiplied by angular velocity), and torque and velocity at peak power were determined by fitting the measured torque and angular velocity values to a rectangular hyperbolic curve (Hill, 1949):

$$V=\frac{\left(F_{\max}+a\right)b}{F+a}-b$$

With F_max_ being maximum isometric torque at 70°, *F* and *V* representing the isotonic load clamp's torque and angular velocity, respectively, and *a* and *b* representing Hill's thermodynamic constants with the units of torque (N•m) and velocity (°/s), respectively. All curve‐fitting was performed in Python 3 using least square error optimization.

### 2.5 Isokinetic conventional resistance training

At the start of each session, the stimulus current was first adjusted to produce maximum isometric torque (pulse duration = 0.1 ms, frequency = 100 Hz, train duration = 500 ms) at an ankle angle of 90°. Maximum isometric torque was recorded for each training session to track changes in muscle strength throughout the training period. The current was then adjusted to produce an isometric torque that as closely as possible matched 70% of maximum isometric torque. This 70% submaximal current was kept constant during both the concentric and eccentric phases of conventional resistance training. The training consisted of 3 sets of 12 repetitions, where each repetition had: (1) a 500‐ms isometric pre‐activation at 70°; (2) shortening from 70° to 110° at 40°/s; (3) lengthening from 110° to 70° at 40°/s; and (4) 1 s rest before the next repetition.

### 2.6 Isokinetic eccentric overload training

Eccentric overload training was performed identically to conventional training. However, the current was increased to evoke a maximal contraction (100% of maximal torque) during only the eccentric phase of each repetition. We adjusted the number of repetitions during eccentric overload training to account for the higher force during the eccentric phase; training consisted of 3 sets of 8 repetitions. To determine the repetition ranges, the sum of the positive work during shortening and absolute negative work while lengthening throughout a concentric‐eccentric contraction was calculated during extensive piloting to ensure that volume was matched as closely as possible between the two training modalities.

### 2.7 Serial sarcomere number determination

Following their final mechanical testing session, rats were sacrificed via isoflurane anesthetization followed by CO_2_ asphyxiation and cervical dislocation. The hindlimbs were amputated, skinned with all muscles overlying the plantar flexors removed, and fixed in 10% phosphate‐buffered formalin with the ankle pinned at 90° (from foot to tibia). After fixation for 1–2 weeks, the soleus and MG were dissected off the lower leg, weighed, then re‐submerged in formalin until the commencement of SSN estimations. To commence the process of SSN estimations, the muscles were rinsed with phosphate‐buffered saline, then digested in 30% nitric acid for ~6 h to remove connective tissue and allow for individual muscle fascicles to be teased out (Butterfield et al., 2005b; de la Tour et al., 1979; Hinks et al., 2022).

For each muscle, two fascicles were obtained from each of the proximal, middle, and distal regions (i.e., *n* = 6 fascicles total per muscle). SSN and FL values were averaged across these six fascicles for the reporting of data. Dissected fascicles were placed on glass microslides (VWR International, USA), then FLs were measured using ImageJ software (version 1.53f, National Institutes of Health, USA) from pictures captured by a level, tripod‐mounted digital camera, with measurements calibrated to a ruler in plane with the fascicles. Sarcomere length (SL) measurements were taken at *n* = 6 different locations proximal to distal along each fascicle via laser diffraction (Coherent, Santa Clara, CA, USA) with a 5‐mW diode laser (~1 mm beam diameter, 635 nm wavelength) and custom LabVIEW program (Version 2011, National Instruments, Austin, TX, USA) for a total of *n* = 36 SL measurements per muscle. For each fascicle, the six SL measurements were averaged to obtain a value of average SL. Given the laser diameter of ~1 mm, one SL measurement itself represents an average of hundreds to thousands of SLs. Our total quantity of SL and FL measurements is consistent with previous studies (Butterfield et al., 2005b; Chen et al., 2020; Hinks et al., 2022; Rilling et al., 2026). The standard deviation (SL SD) is an index of sarcomere length nonuniformity. For each fascicle, the six SL measurements were averaged to obtain a value of average SL. Serial sarcomere number of each fascicle was calculated as:

$$\begin{array}{c} \text{Serial sarcomere number}=\\ \;\text{fascicle length}/\text{average sarcomere length}. \end{array}$$

### 2.8 Determination of physiological cross‐sectional area

To gain further insight on hypertrophy, we calculated physiological cross‐sectional area (PCSA; in cm^2^) using the equation (Chen et al., 2020; Ward & Lieber, 2005):

$$\text{PCSA}=\frac{\text{Muscle mass}\;}{\text{Muscle density}\times \text{normalized}\;\mathrm{FL}}$$

Muscle density was assumed to be 1.112 g/cm^3^ (Ward & Lieber, 2005). Normalized FL was calculated using FL of dissected fascicles and measured SL in the equation (Ward & Lieber, 2005):

$$\text{Normalized}\;\mathrm{FL}=\mathrm{FL}\;\frac{\mathrm{SLo}}{\text{measured}\;\mathrm{SL}}$$

SLo represents optimal SL of rat muscle at rest, assumed to be ~2.7 μm based on previous literature (Chen et al., 2020; Zuurbier et al., 1995).

### 2.9 Sample size determination

Our primary outcome was soleus SSN following resistance training. Based on our previous work comparing conventional and eccentric overload training, which demonstrated a large effect size (Cohen's *d* = 2.7), we estimated that seven animals per group would provide sufficient statistical power to detect a significant difference between training modes. To account for potential attrition and the added variability associated with the OVX intervention, we increased the sample size by three animals per group. Therefore, this resulted in a final target of 10 animals per group.

### 2.10 Statistical analyses and data presentation

**FIGURE 2:**
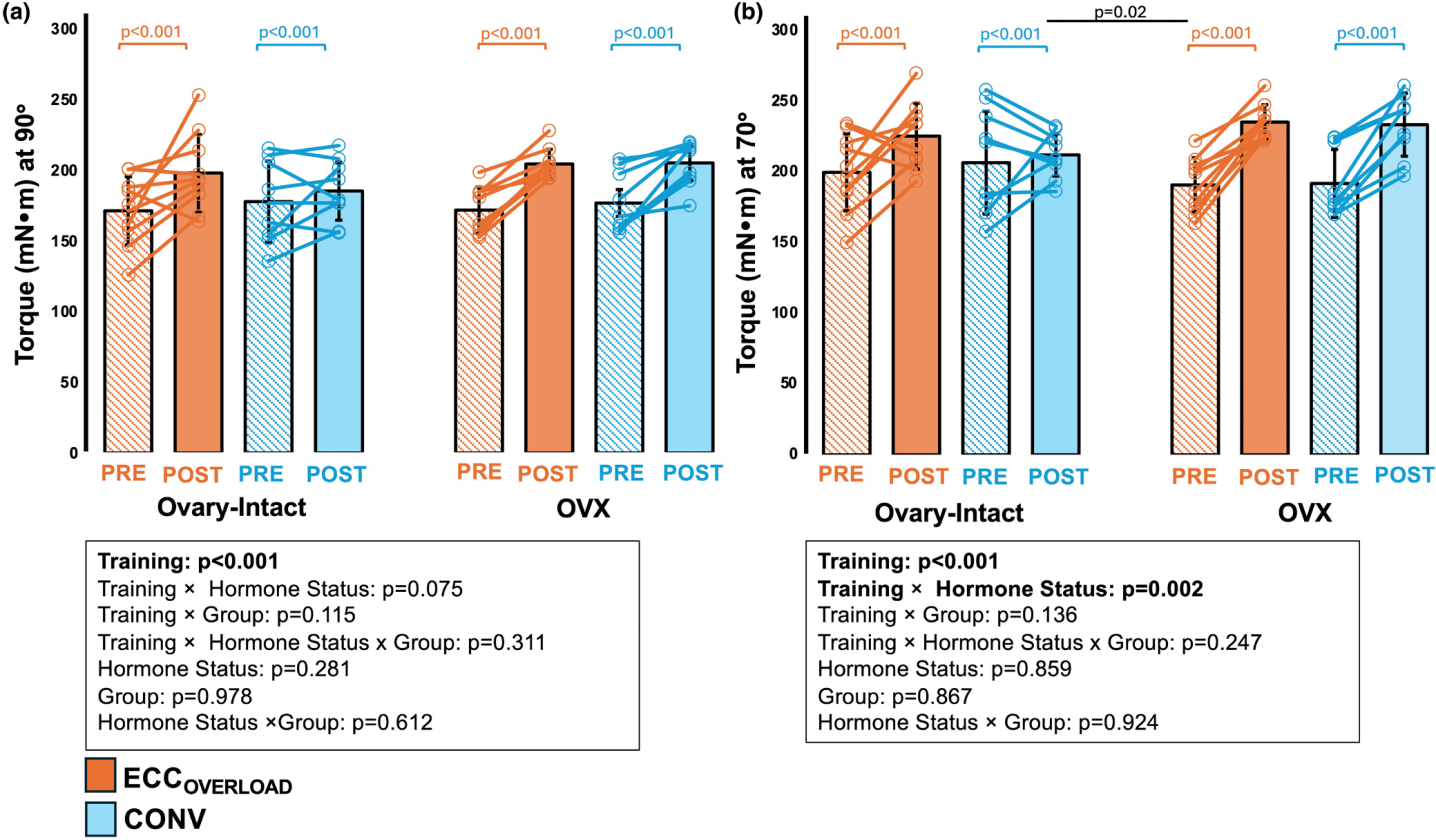
Differences in plantar‐flexor torque with eccentric overload and conventional training in ovary‐intact (*n* = 19) and OVX (*n* = 19) rats at a 90° and 70° ankle angle. (a) Absolute plantar flexor torque pre and post training at 90° for ovary intact and OVX. (b) Absolute plantar flexor torque pre and post training at 70° where the CONV group is shown in blue and the ECC_OVERLOAD_ group is shown in orange. Statistical comparisons were performed using a three‐way repeated measures ANOVA (Group × Training × Hormone Status). Data are displayed as mean ± standard deviation.

**FIGURE 3:**
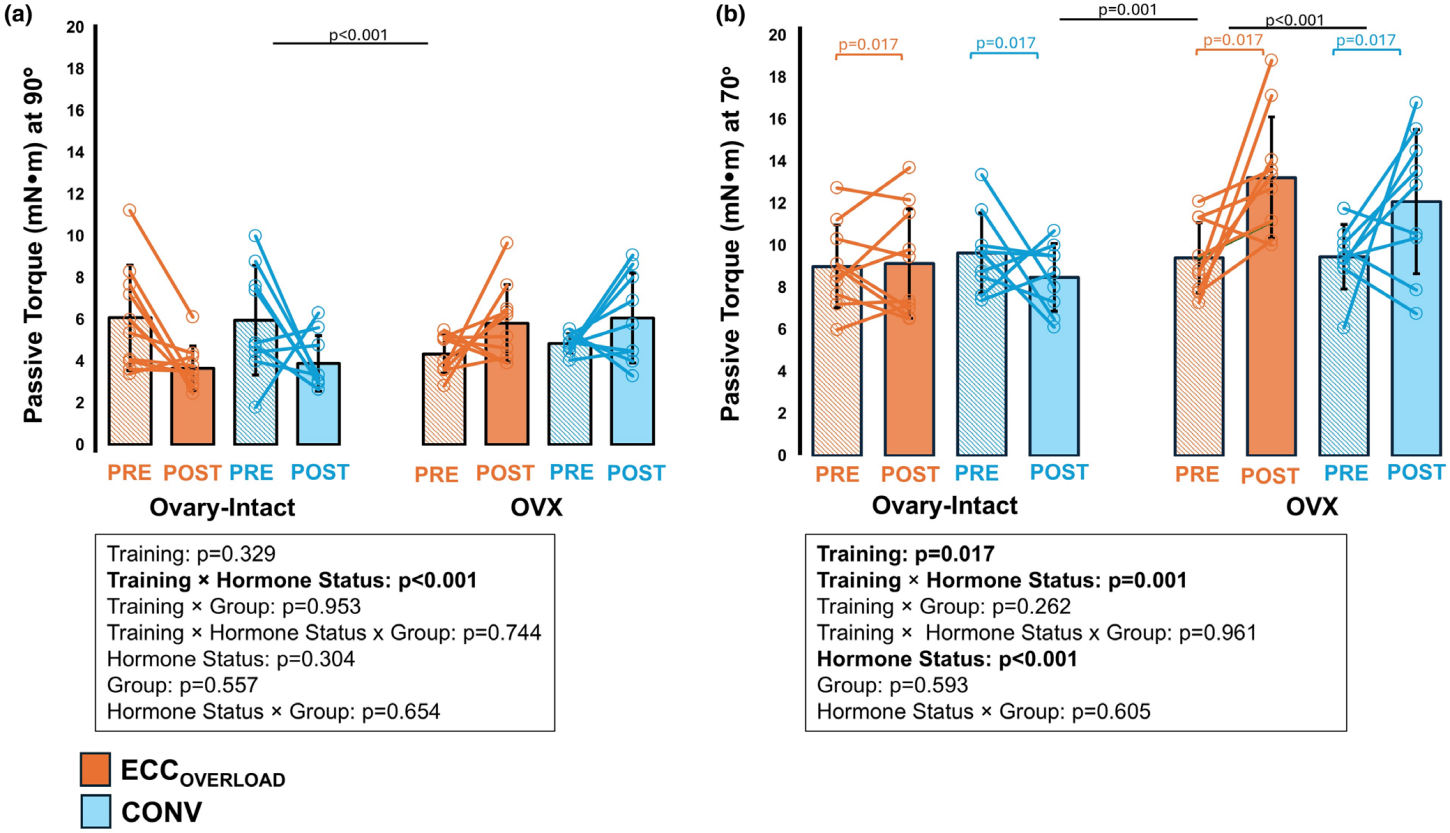
Differences in passive plantar flexor torque in ovary‐intact (*n* = 19) and OVX (*n* = 19) rats with eccentric overload and conventional at a 90° and 70° ankle angle. (a) Passive plantar flexor torque at a 90° ankle angle. (b) Passive plantar flexor torque at a 70° ankle angle where the CONV group is shown in blue and the ECC_OVERLOAD_ group is shown in orange. Statistical comparisons were performed using a three‐way repeated measures ANOVA (Group × Training × Hormone Status). Data are displayed as mean ± standard deviation.

**FIGURE 4:**
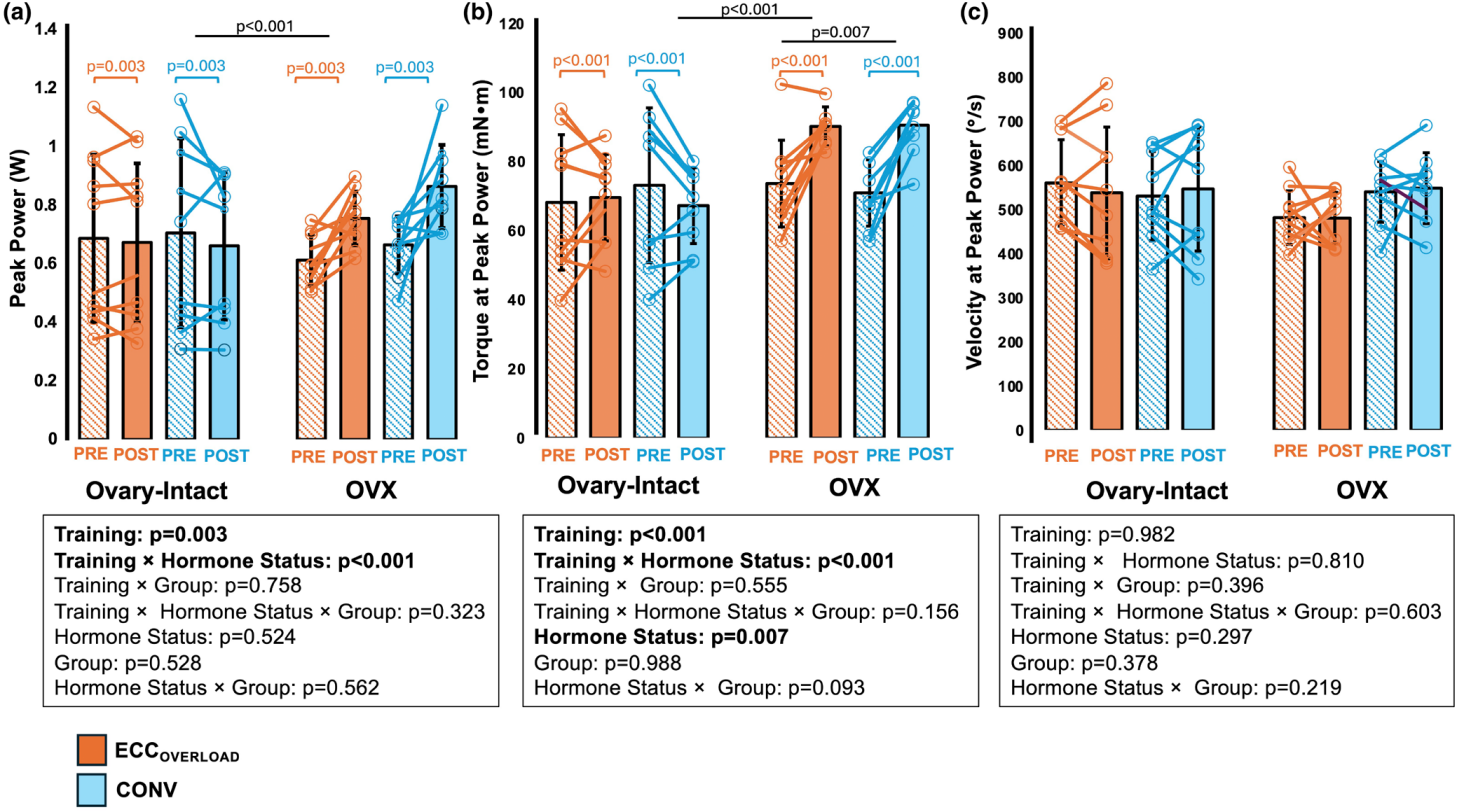
Changes in plantar flexor peak power, velocity at peak power and torque at peak power with training in ovary‐intact (*n* = 19) and OVX rats (*n* = 19). (a) Peak power pre and post training. (b) Changes in torque at peak power with training. (c) Differences in velocity at peak power in response to training. The CONV group is shown in blue, and the ECC_OVERLOAD_ group is shown in orange. Statistical comparisons were performed using a three‐way repeated measures ANOVA (Group × Training × Hormone Status). Data are displayed as mean ± standard deviation.

**FIGURE 5:**
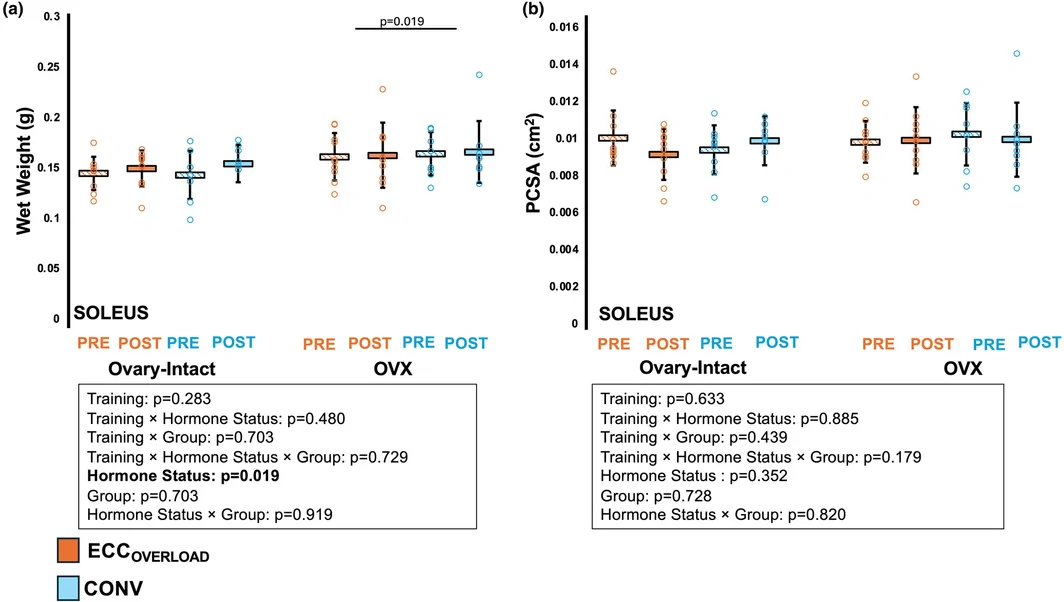
Differences in soleus (a) muscle wet weight and (b) physiological cross‐sectional area (PCSA) pre to post training in ovary‐intact (*n* = 19) and OVX (*n* = 19) rats. The CONV group is shown in blue, and the ECC_OVERLOAD_ group is shown in orange. Statistical comparisons were performed using a three‐way repeated measures ANOVA (Group × Training × Hormone Status). Data are displayed as mean ± standard deviation.

**FIGURE 6:**
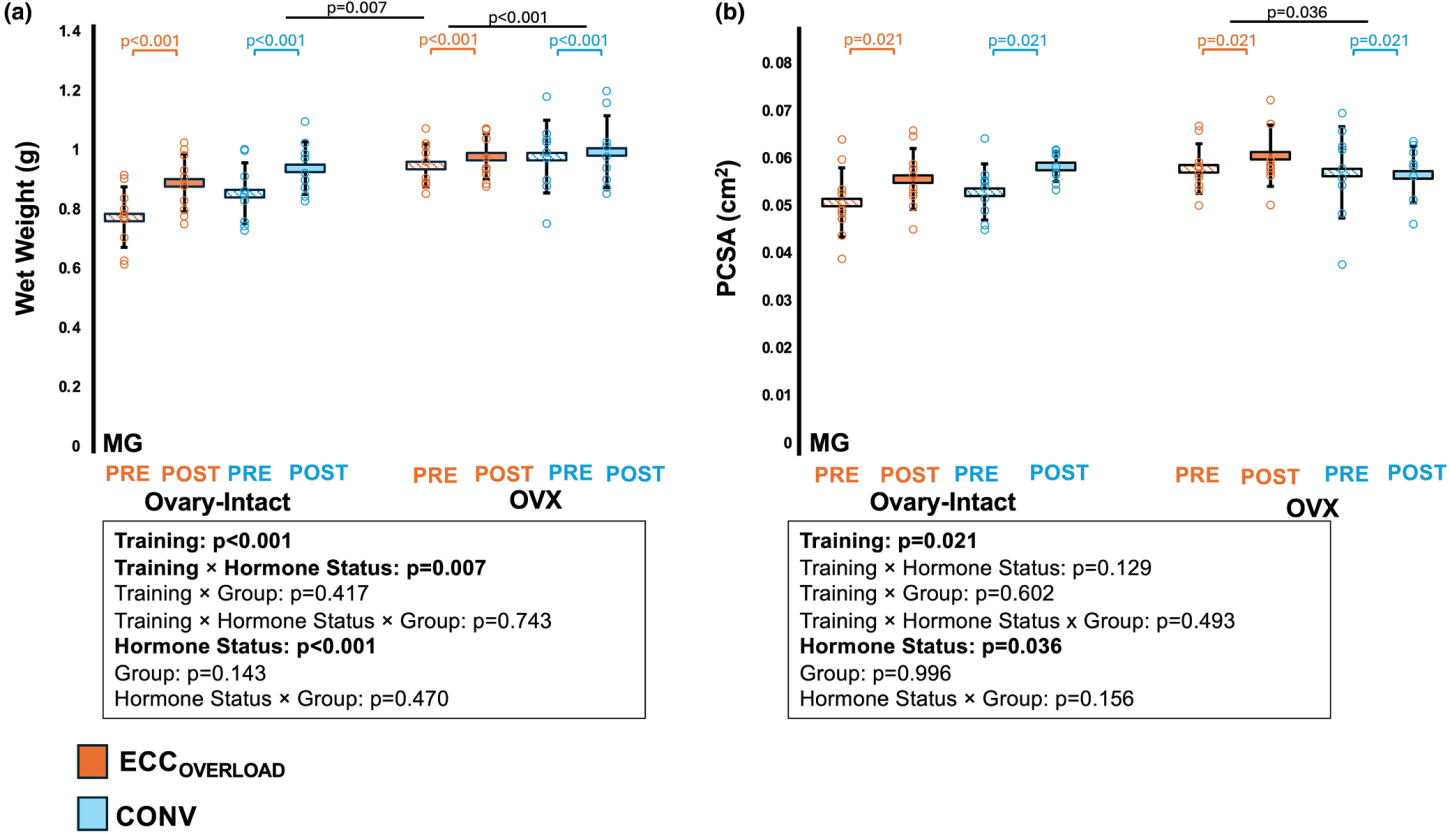
Differences in MG (a) muscle wet weight and (b) physiological cross‐sectional area (PCSA) pre to post training in ovary‐intact (*n* = 19) and OVX (*n* = 19) rats. The CONV group is shown in blue, and the ECC_OVERLOAD_ group is shown in orange. Statistical comparisons were performed using a three‐way repeated measures ANOVA (Group × Training × Hormone Status). Data are displayed as mean ± standard deviation.

**FIGURE 7:**
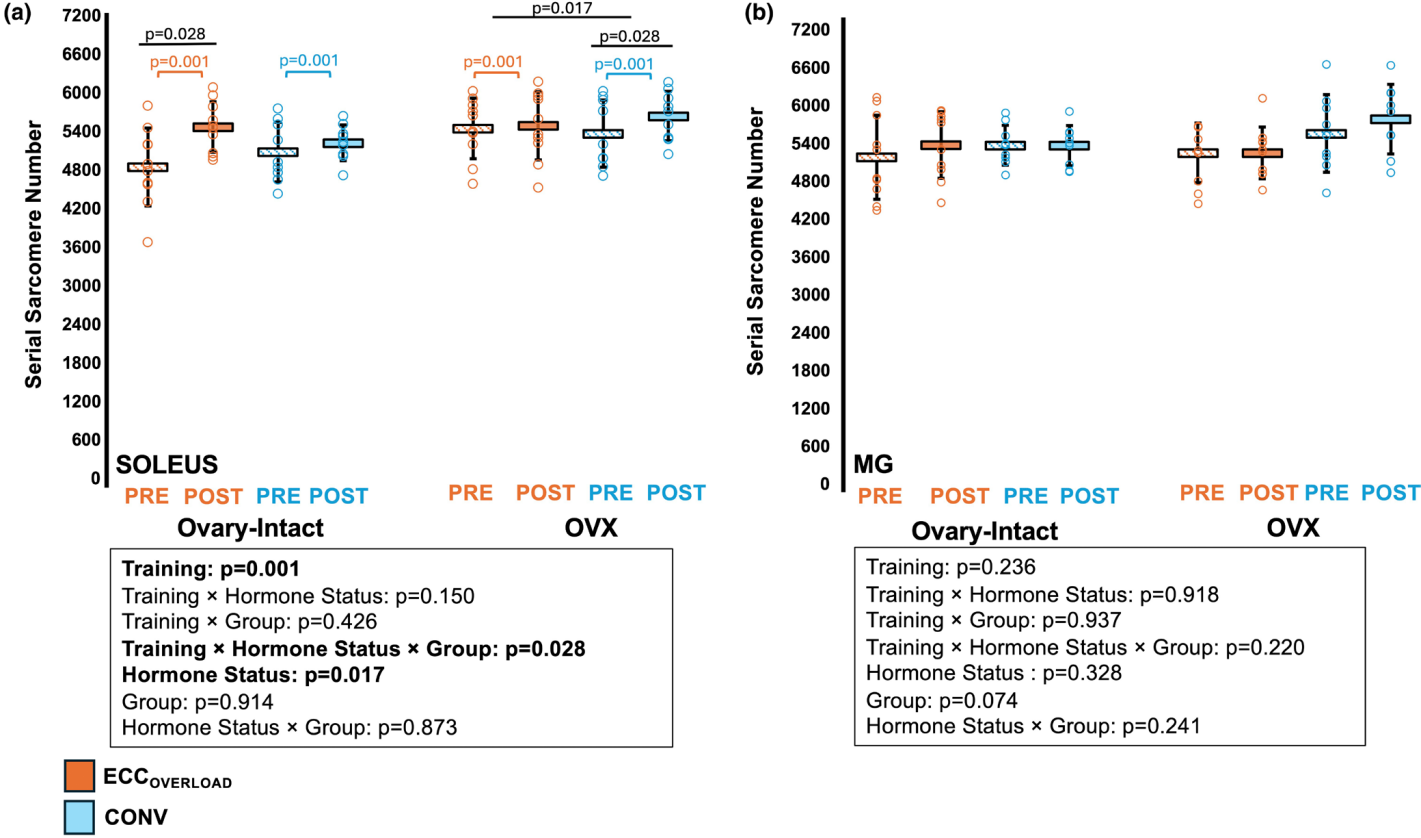
Changes in soleus and MG serial sarcomere number with training in OVX (*n* = 19) and ovary‐intact (*n* = 19) rats. Pre represents the untrained right leg as an internal control and post represents the trained left leg. (a) Differences in soleus serial sarcomere number in the trained (left) and untrained (right) leg in OVX (*n* = 19) and ovary‐intact (*n* = 19) rats. (b) Changes in MG serial sarcomere number with training. The CONV group is shown in blue, and the ECC_OVERLOAD_ group is shown in orange. Statistical comparisons were performed using a three‐way repeated measures ANOVA (Group × Training × Hormone Status). Data are displayed as mean ± standard deviation.

All statistical analyses were performed in SPSS Statistics Premium version 28. Normality of data was confirmed using Shapiro–Wilk tests. Differences in SSN, SL, FL, muscle wet weight, and PCSA between the trained and untrained leg were assessed by a three‐way repeated measures analysis of variance (ANOVA) (Hormone Status [Intact, OVX] × Training Group [CONV, ECC_OVERLOAD_] × Training [Trained leg, Untrained leg]). Differences in peak power, torque, and passive torque were also assessed via three‐way repeated measures ANOVA (Hormone Status [Intact, OVX] × Training Group [CONV, ECC_OVERLOAD_] × Training [pre‐training, post‐training]). Baseline differences were analyzed using a univariant ANOVA with fixed factors of Estradiol Hormone Status [Intact, OVX] and Training Group [CONV, ECC_OVERLOAD_]. For all three‐way ANOVAs, training was treated as a within‐subject factor, hormone status and training group were treated as between‐subject factors. A Greenhouse–Geisser correction for sphericity was applied for all ANOVAs, and a Sidak correction was applied to all pairwise comparisons. Significance was set at *α* < 05 and eta‐squared (*η* ^2^) represents the effect size. For comparisons of pre‐ and post‐training mechanical measurements within the same rat leg, individual data points were connected by lines to indicate the paired nature of the measurements (Figures 2, 3, 4). For morphological comparisons between the trained left leg and untrained right leg within the same rat, individual data points were not connected (Figures 5, 6, 7). All data are presented as mean ± SD.

## 3 RESULTS

### 3.1 Data exclusion

One rat in the CONV intact and one rat in the CONV OVX group experienced health complications precluding them from continuing the study and were excluded from further testing and analysis of mechanical and morphological data. The sample size was adjusted to *n* = 38 (ECC_OVERLOAD_ = 10 intact rats, CONV = 9 intact rats, ECC_OVERLOAD_ = 10 OVX rats, CONV = 9 OVX rats) for all data.

### 3.2 Baseline differences

#### 3.2.1 Pre‐training differences for mechanical outcomes

There were no differences in peak torque at 90° or 70° pre‐training values between training groups (*p* = 0.445; *η* ^2^ = 0.017; *p* = 0.537; *η* ^2^ = 0.011; Figure 2) or OVX/intact (*p* = 0.976; *η* ^2^ = 0). Passive torque at 90° was greater in intact rats compared to OVX at baseline (*p* = 0.027; *η* ^2^ = 0.136; Figure 3) but was not different between training groups (*p* = 0.764; *η* ^2^ = 0.003). At 70° there were no differences in passive torque between training groups (*p* = 0.566; *η* ^2^ = 0.010; Figure 3) or OVX/intact (*p* = 0.849; *η* ^2^ = 0.001). Finally, peak power at baseline was not different between training groups (*p* = 0.631; *η* ^2^ = 0.007; Figure 4a) or between OVX/intact rats (*p* = 0.436; *η* ^2^ = 0.018).

#### 3.2.2 Pre‐training differences for morphological outcomes

Soleus wet weight was greater for OVX as compared with ovary intact rats at baseline (*p* = 0.011; *η* ^2^ = 0.174; Figure 5a) likely owing to greater fat infiltration within the muscle (Jackson et al., 2013). There was no effect of training group at baseline (*p* = 0.918; *η* ^2^ = 0). Additionally, soleus PCSA did not differ between OVX and ovary intact rats (*p* = 0.497; *η* ^2^ = 0.014) or between training groups (*p* = 0.803; *η* ^2^ = 0.002). Soleus SSN was higher in the OVX group (*p* = 0.013; *η* ^2^ = 0.168) but did not differ between training groups (*p* = 0.655; *η* ^2^ = 0.006).

MG wet weight (*p* < 0.001; *η* ^2^ = 0.378; Figure 6a) and PCSA (*p* = 0.021; *η* ^2^ = 0.147; Figure 6b) were greater in the OVX group compared to the ovary intact group at baseline with no differences between training groups (*p* = 0.767; *η* ^2^ = 0.003). MG SSN did not differ between OVX/intact rats (*p* = 0.467; *η* ^2^ = 0.016) or between training groups (*p* = 0.164; *η* ^2^ = 0.056; Figure 7b).

### 3.3 Mechanical adaptations

#### 3.3.1 Peak torque at a 90‐ and 70‐degree ankle angle

Peak torque at 90° increased ~16% in ECC_OVERLOAD_ intact rats, ~4% in CONV intact rats, ~19% in ECC_OVERLOAD_ OVX rats and ~16% in CONV OVX rats respectively demonstrating an effect of training (*p* < 0.001; *η* ^2^ = 0.555). There were no differences between training groups (*p* = 0.978; *η* ^2^ = 0) or OVX/intact rats (*p* = 0.281; *η* ^2^ = 0.034). At 70°, there was an interaction of hormone status × training (*p* = 0.002; *η* ^2^ = 0.248) where OVX rats had a greater overall increase in torque with training as peak torque increased ~12% in ECC_OVERLOAD_ intact rats, ~1% in CONV intact rats, ~23% in ECC_OVERLOAD_ OVX rats and ~ 22% in CONV OVX rats. Additionally, there was an effect of training (*p* < 0.001; *η* ^2^ = 0.588).

#### 3.3.2 Passive torque at a 90‐ and 70‐degree ankle angle

Passive torque at 90° decreased ~40% in ECC_OVERLOAD_ intact rats and ~50% in CONV intact rats. Conversely, passive torque increased ~34% in ECC_OVERLOAD_ OVX rats and ~25% in CONV OVX rats demonstrating an interaction of training × hormone status (*p* < 0.001; *η* ^2^ = 0.318) driven by the decrease in passive torque in intact rats and increase in OVX rats. There was no effect of training (*p* = 0.329; *η* ^2^ = 0.028) or training group (*p* = 0.557; *η* ^2^ = 0.01). Passive torque at 70° showed an interaction of training × hormone status (*p* = 0.001; *η* ^2^ = 0.261) due to increases in passive tension in OVX rats with training. There was an overall decrease of ~5% in ovary‐intact rats compared to an ~35% increase in OVX rats resulting in an effect of training (*p* = 0.017; *η* ^2^ = 0.156). Additionally, there was an effect of hormone status (*p* < 0.001; *η* ^2^ = 0.302).

#### 3.3.3 Peak power, torque at peak power, and velocity at peak power

Across both ECC_OVERLOAD_ and CONV training peak power decreased ~4% in intact rats and increased ~27% in OVX rats resulting in an interaction of training × hormone status (*p* < 0.001; *η* ^2^ = 0.383; Figure 4a). Additionally, there was an overall effect of training (*p* = 0.03; *η* ^2^ = 0.239; Figure 4a) such that power increased overall with training; however, the training group did not impact the overall increase.

Torque at peak power decreased ~3% in ovary‐intact rats and increased ~25% in OVX rats. There was an interaction of training × hormone status (*p* < 0.001; *η* ^2^ = 0.488; Figure 4b) and an overall training effect (*p* < 0.001; *η* ^2^ = 0.367). Comparatively, velocity at peak power showed no training × hormone status interaction (*p* = 0.81; *η* ^2^ = 0.002) or effect of training (*p* = 0.982; *η* ^2^ = 0), indicating the increases in peak power were likely primarily driven by increased torque production at a maintained velocity.

### 3.4 Morphological adaptations

#### 3.4.1 Wet weight and physiological cross‐sectional area

Soleus wet weight had an effect of hormone status (*p* = 0.019; *η* ^2^ = 0.151; Figure 5a) where it was greater in OVX rats as compared with intact rats likely owing to greater fat infiltration within the muscle (Jackson et al., 2013). There were no interactions, effect of training (*p* = 0.283; *η* ^2^ = 0.034) or training group (*p* = 0.703; *η* ^2^ = 0.004). Soleus PCSA had no effect of hormone status (*p* = 0.352; *η* ^2^ = 0.026; Figure 5b) and no effect of training (*p* = 0.633; *η* ^2^ = 0.007) or training group (*p* = 0.728; *η* ^2^ = 0.004).

MG wet weight had an interaction of training × hormone status (*p* = 0.007; *η* ^2^ = 0.198; Figure 6a) due to an increase in wet weight in response to training in ovary‐intact rats. There was an overall effect of training (*p* < 0.001; *η* ^2^ = 0.388) and hormone status (*p* < 0.001; *η* ^2^ = 0.296) as there was a higher overall wet weight in OVX rats. MG PCSA showed an overall effect of training (*p* = 0.021; *η* ^2^ = 0.147) and of hormone status (*p* = 0.036; *η* ^2^ = 0.123; Figure 6b) due to a greater PCSA in OVX rats.

#### 3.4.2 Soleus adaptations in serial sarcomere number

There was an interaction of training × hormone status × group (*p* = 0.028; *η* ^2^ = 0.135; Figure 7a) where the ECC_OVERLOAD_ ovary intact females and the CONV OVX were the greatest responders. Soleus serial sarcomere number derived from FL and SL (Figure S1) increased with training ~13% in the ECC_OVERLOAD_ group and ~3% in the CONV group in ovary‐intact rats. Comparatively, SSN increased ~1% in the ECC_OVERLOAD_ group and ~5% in the CONV group in OVX rats (*p* < 0.001; *η* ^2^ = 0.285) showing an overall effect of training (*p* = 0.001; *η* ^2^ = 0.285, Figure 7a).

#### 3.4.3 MG adaptations in serial sarcomere number

SSN derived from FL and SL (Figure S2) in the MG had no change in the ECC_OVERLOAD_ and CONV group in intact and OVX rats, resulting in no effect of training (*p* = 0.236; *η* ^2^ = 0.041; Figure 7b). There was no effect of group (*p* = 0.074; *η* ^2^ = 0.091) nor interactions between training × hormone status × training group (*p* = 0.220; *η* ^2^ = 0.044; Figure 7b).

## 4 DISCUSSION

We investigated the effects of ovariectomy and its associated changes in the hormonal milieu (e.g., estradiol deficiency) on the muscle's response to ECC_OVERLOAD_ and CONV training. Intact and OVX rats completed 4 weeks of ECC_OVERLOAD_ or CONV training and then the soleus and medial gastrocnemius muscles were harvested for quantification of serial sarcomere number. Longitudinal muscle remodeling was modulated by hormone status where for the soleus, intact rats were the greatest responders to ECC_OVERLOAD_ training (~13%) compared to CONV trained intact (~3%) rats and both ECC_OVERLOAD_ (~1%) and CONV trained OVX rats (~5%). Thus, estradiol deficiency and associated changes in the hormonal milieu may confer a blunted adaptation in longitudinal muscle remodeling in response to ECC_OVERLOAD_ training.

### 4.1 Changes to the hormonal milieu of OVX rats modulates longitudinal muscle remodeling

The extent of longitudinal muscle remodeling differed in intact and OVX rats highlighting the potential role of changes to the hormonal milieu, in particular, estradiol in response to training. In the estradiol deficient state there was a blunted adaptation to ECC_OVERLOAD_ training. Chronic eccentric exercise training is known to promote longitudinal muscle remodeling (Butterfield et al., 2005a; Hinks, Vlemmix, & Power, 2024; Wagle et al., 2017). That said, chronic eccentric exercise training can also result in damage to the muscle (exercise‐induced muscle damage (EIMD)) (Proske & Morgan, 2001), which is evident through z‐line streaming and increased inflammation (Proske & Morgan, 2001; Takekura et al., 2001). When incurred, this EIMD may hinder the successful longitudinal remodeling of the muscle.

However, as previously shown, estradiol can have positive effects related to reducing EIMD by facilitating the recovery and repair processes in the muscle. In fact, it can mediate initial damage to the muscle by attenuating leukocyte (i.e., neutrophil) infiltration (Tiidus, 2003), which can accumulate in the muscle releasing cytotoxins (You et al., 2022). It may also offer a protective effect through greater strong binding myosin (Moran et al., 2007) and regulatory light chain (RLC) phosphorylation (Colson et al., 2015; Lai et al., 2016) which improves contractile function of the muscle (Phillips et al., 1993) by enhancing the specific force the muscle can produce (Collins, Laakkonen, & Lowe, 2019). Estradiol is also implicated in regulation, activation and proliferation of satellite cells which may accelerate repair processes following damaging exercise (Collins, Arpke, et al., 2019; Enns & Tiidus, 2008; Seko et al., 2020) promoting longitudinal muscle growth.

Current evidence on EIMD points to differences in recovery and muscle repair between OVX and intact rodents. McClung et al. (2007) observed intact and OVX rats' soleus muscle response to disuse atrophy through 10 days of hindlimb suspension. Dystrophin protein disruption (marker of membrane breakdown), creatine kinase (CK), and neutrophil infiltration were analyzed 1‐, 3‐, and 7‐days post‐suspension. Initially, there was no difference in dystrophin or CK levels, indicating initial muscle damage was similar between groups. However, at Day 3, neutrophil levels were 43% higher in the OVX group, and pro‐inflammatory macrophages were still evident at Day 7. Therefore, estradiol deficiency caused an increased and prolonged inflammatory response to muscle damage (McClung et al., 2007).

Similarly, Kosir et al. (2015) used a protocol of 150 eccentric contractions in intact and OVX mice. At Day 3 post protocol, MAC‐1, an inflammatory marker, was higher in OVX rats. Interestingly, torque was maintained better in the OVX group compared to the intact group throughout the eccentric contractions but was blunted 14 days following, suggesting no differences in muscle contractile function but impaired remodeling post damage (Kosir et al., 2015). Therefore, while muscle damage and neutrophil infiltration were not measured in the present study, our ECC_OVERLOAD_ training likely induced muscle damage which was attenuated in intact rats due to protective (Collins, Laakkonen, & Lowe, 2019; Colson et al., 2015; Lai et al., 2016; Moran et al., 2007; Phillips et al., 1993), and reparative (Collins, Arpke, et al., 2019; Seko et al., 2020) effects of estradiol allowing a robust increase in SSN (+~13%). Comparatively, ovariectomy and the estradiol deficient state (in addition to other changes in the hormonal milieu) may have incurred more damage and inflammation to the muscle, blunting the adaptive repair process in response to ECC_OVERLOAD_ (+~1%).

CONV training, however, likely experiences less EIMD owing to the lower intensity eccentric contractions and allowed for reparative processes in the CONV OVX rats enabling a ~5% response to training, like the response seen in intact rats (~3%). Hinks, Vlemmix, & Power (2024) found that in aged rats submaximal eccentric training resulted in a ~11% increase in SSN, however, maximal eccentric training caused a reduction in SSN (Hinks, Patterson, et al., 2024). The maximal stimulus likely caused muscle damage inducing a maladaptive response to training in aged rodents like the blunted response seen in ECC_OVERLOAD_ trained OVX rats. Similarly, Vidal et al. (2020) found sarcomerogenesis was blunted in OVX rats following a strenuous and repetitive stretching protocol. They reported a non‐significant (potentially limited by sample size) increase of ~15%–20% in sarcomerogenesis in ovary‐intact rats and a negligible increase in SSN in OVX rats (Vidal et al., 2020) indicating blunted remodeling in response to intense and chronic stretching in the absence of estradiol. Ultimately, estradiol's protective effect and attenuation of muscle damage may have enhanced the response to ECC_OVERLOAD_ in ovary‐intact rats whereas in the estradiol deficient state a submaximal eccentric stimulus may be more conducive to longitudinal muscle remodeling.

### 4.2 ECC_OVERLOAD_ training is a more optimal stimulus in intact rats

ECC_OVERLOAD_ training in intact rats resulted in an ~13% increase in SSN compared to an ~3% increase for CONV intact rats. ECC_OVERLOAD_ training increases mechanical stress, the force per area of the muscle, beyond that of CONV training while both lengthening the muscle. Consequently, the greater stimulus promotes longitudinal muscle growth by increasing force per cross‐sectional area while activating the muscle to a long length.

The high forces at long muscle lengths are the primary driver of longitudinal sarcomerogenesis (Power et al., 2026) while the increased load throughout the range of motion provides an additional stimulus for muscle remodeling beyond that observed in CONV training. It is important to note here that both training modalities involved an isometric pre‐activation at long muscle lengths at an electrical current used to evoke 70% maximal tetanic force, a shortening contraction at that same current intensity, and then that same intensity for active lengthening (CONV) or a maximal (ECC_OVERLOAD_). Therefore, the intensity of the eccentric contraction, in and of itself, should be regarded as the driver of longitudinal muscle remodeling, and the presence or absence of estradiol dictates the recovery and adaptation from this added stimulus.

### 4.3 Mechanical and morphological discrepancies in torque

Torque increases similarly at the 90° ankle angle with training in ovary‐intact and OVX rats. As well, there was an interaction of training × hormone status, such that there was a larger increase in torque in response to training in OVX rats at 70° (longer muscle length), despite the greatest morphological (soleus SSN) adaptations in intact ECC_OVERLOAD_ trained rats. Likely, this finding highlights that changes at the fascicle level of the muscle may not fully translate to the muscle tendon unit. Alterations in connective tissue, extracellular matrix remodeling, and pennation angle (Aagaard et al., 2001; Hinks, Vlemmix, & Power, 2024; Stone, 1988) may impact torque transmitted from the muscle tendon unit. Additionally, our morphological outcomes focused on the soleus and MG muscle. However, the plantar flexors are composed of the soleus, MG, lateral gastrocnemius, and plantaris (Mansfield & Neumann, 2019). The response of the entire muscle group was not studied and therefore may have adapted differently, contributing to changes in mechanical performance. Overall, alterations at the fiber and fascicle level may not translate to changes in force production by the plantar flexors as a muscle group.

### 4.4 Passive tension decreases in intact rats and increases in OVX rats

Reduced passive tension in the ovary‐intact rats may have been due to the increases in SSN allowing fewer overstretched sarcomeres and thus more optimal SLs at the longer muscle lengths tested (Herzog, 2018). Additionally, although beyond the scope of the present study, eccentric exercise results in remodeling of the extracellular matrix (Hyldahl et al., 2015). Extracellular matrix remodeling following eccentric exercise typically increases stiffness (Kjær et al., 2006), which was likely overcome by the increases in SSN in the ECC_OVERLOAD_ intact rats or the decreased tendon stiffness in intact rats from estradiol (Torricelli et al., 2013). Comparatively, the increases in passive tension in OVX may have been caused by fibrotic remodeling of the ECM or tendon differences in the estradiol deficient state may have promoted a stiffer tendon (Circi et al., 2009) (discussed further below), which heightened overall passive tension of the muscle tendon unit.

### 4.5 Differences in peak power, velocity, and torque at peak power following training

Peak power increased greater in OVX compared to intact rats despite no changes in velocity at peak power. Thus, the increase in peak power was driven by greater increases in torque at peak power in the OVX as compared with the intact group. Although not measured in the present study, changes to tendon compliance, such as decreased tendon elasticity and increased tendon stiffness in the estradiol deficient state (Bridgeman et al., 2010; Circi et al., 2009; Torricelli et al., 2013) may have altered MTU force transmission (Brumitt & Cuddeford, 2015; Lieber & Ward, 2011; Lorimer & Hume, 2016). This requires further investigation.

### 4.6 Additional considerations on hormonal influences on muscle remodeling in the OVX model

Progesterone is a prominent female sex hormone which also declines with ovariectomy. Notably, progesterone supplementation alone in post‐menopausal females did not alter peak torque production (Peters & Burrows, 2006), but can upregulate muscle protein fractional synthesis rates compared to controls (Smith et al., 2014). However, muscle breakdown was not assessed indicating that while progesterone may enhance synthesis, its net effect on muscle growth was not clear. That said, progesterone may aid estradiol on the conservation of the satellite cell pool (Collins, Arpke, et al., 2019) aiding in the growth and repair of muscle. After an ovariectomy, there is a decline in testosterone production from ~0.17 ng/mL to 0.07 ng/mL (Bonilla‐Becerra et al., 2017). Testosterone may impact muscle remodeling through enhanced muscle protein synthesis (MPS). That said, most research on the effects of testosterone on muscle remodeling is in males where testosterone is 10x more potent. Female endogenous levels may not reach a functional threshold to enhance MPS (Alexander et al., 2022). Additionally, with ovariectomy, there is an increase in FSH and LH (Yen & Tsai, 1971) however, there is limited data to suggest that LH and FSH mediate or inhibit longitudinal muscle remodeling, making them an unlikely contribution to the observed adaptations (Ke et al., 2022). While there is a notable decline in testosterone and progesterone, changes may influence muscle remodeling, levels of estradiol can change from ~50–35 pg/mL to < 5 pg/mL (undetectable) following ovariectomy, making the change in estradiol more prominent (Chang et al., 2019). Estradiol re‐supplementation alone in OVX rats has been shown to modulate skeletal muscle recovery from 10 days of hindlimb suspension and disuse atrophy in female rats, restoring cross‐sectional area and muscle mass (McClung et al., 2006) to the levels of intact rats. Therefore, estradiol supplementation alone can facilitate muscle remodeling and is likely responsible for the results of the present study.

### 4.7 Methodological considerations and future directions

Differences in adaptation between the soleus and MG are likely a result of the bi‐articular nature of the MG (Hsu & Chang, 2025) which was not optimized for maximal stretch magnitude. Thus, the MG likely underwent less excursions and/or overall lengthening than the soleus muscle resulting in insignificant increases in SSN. Chronic or repetitive lengthening of a muscle is likely a key driver of sarcomerogenesis. Both groups experienced activation at long lengths; however, the MG crossed both the knee and ankle. The knee angle was fixed at ~150 degrees; therefore, less stretching and lengthening occurred than if it was set to a straight leg position. The shorter muscle length likely favored radial growth, driving the observed increases in PCSA and wet weight. Nonetheless, the MG increased similarly in the ECC_OVERLOAD_ group to what has been previously observed in our lab (Rilling et al., 2026).

There was an interaction of training × training group in soleus SL (Figure S1) where ECC_OVERLOAD_ had larger increases in SL and an effect of group in MG SL (Figure S2), due to greater overall SL in the ECC_OVERLOAD_ group which was likely a result of muscle fixation or a delayed training adaptation. After dissection, the legs are fixed at a 90° ankle angle. Any variation in this fixation or shifts in the muscles L_0_ would cause sarcomere length to be slightly compressed or stretched. Additionally, any late responders may have increases in SL prior to increases in SSN. Some rats may have been adapting still at the time of dissection and fixation. However, this stretch or compression would also occur at the fascicle length. Thus, as we equate SL to FL for SSN, SSN remains a true indicator of longitudinal remodeling.

## 5 CONCLUSION

ECC_OVERLOAD_ training results in greater longitudinal remodeling in ovary‐intact compared to OVX rats mediated by estradiol. Despite greater SSN increases in ovary‐intact ECC_OVERLOAD_ trained rats, there were no differences in torque production at shorter muscle lengths between groups. However, passive tension was lower in ovary‐intact as compared to OVX rats. These findings highlight the role of estradiol in the adaptive process of muscle following exercise training.

## AUTHOR CONTRIBUTIONS

**Amelia Rilling:** Conceptualization; data curation; formal analysis; investigation; methodology. **Alexandra Q. Kirkup:** Investigation; methodology. **Avery Hinks:** Investigation; methodology. **Martino V. Franchi:** Investigation. **Geoffrey A. Power:** Conceptualization; data curation; funding acquisition; investigation; methodology; project administration; supervision.

## FUNDING INFORMATION

This project was supported by the Natural Sciences and Engineering Research Council of Canada (NSERC), grant number RGPIN‐2024‐03782.

## CONFLICT OF INTEREST STATEMENT

Dr. Geoffrey Power is an Associate Editor at Physiological Reports and was blinded from reviewing or making decisions for the manuscript. Another Editor oversaw the manuscript process for this article.

## ETHICS STATEMENT

Approval was given by the University of Guelph's Animal Care Committee and all protocols followed CCAC guidelines (AUP #4905).

## Supporting information


Figures S1–S2.

## Data Availability

All data generated or analyzed during the study are available from the corresponding author upon request.
