# Supplementary material for: Serial sarcomere adaptation to eccentric overload training is blunted in ovariectomized rats
Source: Physiol Rep. 2026 Aug 24;14(16):e71070. doi: 10.14814/phy2.71070 (PMC13501099; doi:10.14814/phy2.71070)
Supplement: Supplementary file 1 [file PHY2-14-e71070-s001.docx]

**Supplemental Figures**

**Supplemental Figure 1: Changes in soleus A) fascicle length (mm) and B) sarcomere length (µm)** in response to ECCOVERLOAD or CONV resistance training in ovary intact (n=19) or OVX (n=19) rats. The CONV group is shown in blue, and the ECC_OVERLOAD_ group is shown in orange. Statistical comparisons were performed using a three-way repeated measures ANOVA (Group × Training × Hormone Status). Data are displayed as mean ± standard deviation. **Results**: Soleus FL showed an interaction of training × hormone status (p=0.005; η² =0.206) due to greater increases in intact rats and an interaction of training × group (p=0.032; η² =0.128) driven by an increased overall response to ECC_OVERLOAD._ FL increased ~16% in the ECC_OVERLOAD_ group and ~5% in the CONV group in intact females. In the OVX females FL increased ~2% in the ECC_OVERLOAD_ group and ~1% in the CONV group demonstrating an effect of training (p<0.001; η² =0.330) and an effect of hormone status (p=0.029; η² =0.133) with no effect of training group (p=0.806; η² =0.002). Soleus SL had an interaction of training × group (p=0.033; η² =0.127) as a likely result of fixation or late responding sarcomeres in the ECC_OVERLOAD_ group which are slightly stretched prior to increases in SSN. There was no effect of training (p=0.304; η² =0.031), no effect of training group (p=0.625; η² =0.007) and no effect of hormone status (p=0.296; η² =0.032).

**Supplemental Figure 2: Differences in MG fascicle length (mm) and sarcomere length (µm)** in response to ECCOVERLOAD or CONV resistance training in ovary intact (n=19) or OVX (n=19) rats. The CONV group is shown in blue, and the ECC_OVERLOAD_ group is shown in orange. Statistical comparisons were performed using a three-way repeated measures ANOVA (Group × Training × Hormone status). Data are displayed as mean ± standard deviation. **Results:** MG FL had an ~5% increase in ECC_OVERLOAD_ and an ~5% increase CONV intact rats. In OVX rats there was an ~1% decrease in ECC_OVERLOAD_ and an ~3% increase in CONV with no overall effect of training (p=0.056; η² =0.103; Figure 2a). There was no training × hormone status (p=0.139; η² =0.063), hormone status × group (p=0.761; η² =0.003), training × group (p=0.453; η² =0.017) or training × hormone status × group (p=0.467; η² =0016) interactions. MG SL differed between training groups (p=0.007; η²=0.193) but did not differ between OVX/intact (p=0.118; η² =0.070) or with training (p=0.480; η²=0.015).
